# Medical Opinion and Movement

**Published:** 1907-04-06

**Authors:** 


### Medical Opinion and Movement.

The "white line" is an interesting phenomenon which was first made known to French physicians by M. Sergent. It is the converse of the meningeal streak, and is produced by drawing the finger-nail across the skin of the patient's abdomen. It appears in about 30 to 60 seconds, and lasts from two to five minutes. It is supposed to be indicative of low arterial tension, and occurs in the specific fevers, septicæmia, and exhaustion from over-exertion. It has also been found in Addison's disease, and M. Sergent suggests that the low tension giving rise to the phenomenon is due to some alteration in the cells of the suprarenal capsules. Administration of suprarenal extract causes a disappearance both of the "white line" and also of the low tension. The connection between the phenomenon and disease of the suprarenal capsules has been further confirmed by MM. Seridey and Tinel, who have recently reported a case of tuberculous meningitis, in which the meningeal streak was replaced by the "white line." The necropsy revealed meningeal, pulmo~ary, and peritoneal miliary tuberculosis, and large tubercles in the suprarenal capsules. In such cases, therefore, the "white line" may prove an important indication of involvement of these organs.

It is with regret that we have to record the death of Germany's most famous surgeon, Ernst von Bergmann. Only recently he celebrated his seventieth birthday, and the congratulations and good wishes he received from all parts on that occasion showed the esteem in which he was held by his fellow-countrymen. For the last few years he had suffered from intestinal trouble, and having eventually himself made a diagnosis of cancer, he decided to undergo operation. Unfortunately he succumbed a few hours after the operation from heart failure. Professor von Bergmann was a strong personality of striking appearance. He was born, a Russian subject, in Livonia, but was of German parentage. He served on the Russian medical staff in the wars of 1866 and 1870-1, and later he acted as consulting surgeon to the Russian Danube force in the campaign of 1877. He was Professor of Surgery at Dorpat University, and later in Berlin. As surgeon to the Emperor Frederick he was the first to recognise the disease from which his Majesty was suffering as cancer, and he is supposed to have inspired the attacks on Sir Morell Mackenzie after the death of the Emperor. He was frequently called to St. Petersburg in consultation for members of the Imperial family, and recently he was summoned to Constantinople by the Sultan. He was renowned for his abruptness of manner and speech combined with a rough good humour, and many anecdotes are told of him in this respect.

In spite of the emphatic and oft-reiterated opinion of fever authorities in favour of the administration of antitoxin at the earliest possible moment in cases of diphtheria, there is reason to believe that it is still a common practice among medical men to await the result of the bacteriological examination of the swab before giving the first injection. This means a delay of at least 24 hours. Such a delay is undoubtedly sufficient in some cases to determine a fatal issue to the attack, and in many cases prevents that rapid abortion of the disease which is so striking when the injections are made within the first few hours of onset. Dr. Bolton, in a recent paper on the treatment of acute heart failure, draws attention to the importance of early injections of the diphtheria antitoxin, so as to arrest the effects of the diphtheria poison on the heart. He points out that acute diphtheria poisoning kills the patient by the direct action of the toxin on the heart, and also on the vagus nucleus, causing slowness and irregularity of the heart's action and a tendency to sudden syncope. The efficiency of the antitoxin depends entirely upon the stage of the disease at which it is given and upon the size of the dose. If antitoxin is given on the first day of the disease the mortality is only ½ per cent., and this mortality increases in exact proportion to the delay in its administration. Experiments on animals and clinical experience teach that the dose of antitoxin must be large to be efficacious. In mild cases 4,000 to 6,000 units should be injectd at once, and in severe cases as much as 24,000 units. If the membrane shows signs of spreading, the dose should be repeated on the following day, but the important point is to get a sufficiently large dose into the patient at the earliest possible moment.

In face of the overloaded condition of the legislative machine in other directions, there does not seem much prospect that any headway will be made with measures of public health during the present session of Parliament. Such measures are of little concern to the general public, and they do not, therefore, count as assets for electioneering purposes. Consequently they evoke little enthusiasm, and are among the first to be thrown overboard when the ship is too full. There are two Bills now before Parliament, and down for a second reading this month, dealing with the question of tenure of office of medical officers of health, and seeking to prevent arbitrary dismissal by the local authorities by securing a right of appeal to the Local Government Board. This is by no means their first appearance, and will certainly not be the last, at least for one of them. The Parochial Medical Officers Bill for Scotland has an influential backing on both sides of the House, and has therefore some prospect of becoming law. The Public Health Officers Bill, however, which refers only to England, has been blocked on behalf of hostile municipal bodies. This is much to be regretted in the interests of public health. So long as medical officers of health are dependent for their livelihood upon the goodwill of the local authority, it cannot be expected that they will recommend or put into execution measures which, though necessary for the public welfare, may run counter to the interests of the individual members of the council.

